# Using novel data and ensemble models to improve automated labeling of Sustainable Development Goals

**DOI:** 10.1007/s11625-024-01516-3

**Published:** 2024-07-24

**Authors:** Dirk U. Wulff, Dominik S. Meier, Rui Mata

**Affiliations:** 1 Max Planck Institute for Human Development, Berlin, Germany; 2 University of Basel, Basel, Switzerland

**Keywords:** SDGs, Natural language processing, Machine learning

## Abstract

A number of labeling systems based on text have been proposed to help monitor work on the United Nations (UN) Sustainable Development Goals (SDGs). Here, we present a systematic comparison of prominent SDG labeling systems using a variety of text sources and show that these differ considerably in their sensitivity (i.e., true-positive rate) and specificity (i.e., true-negative rate), have systematic biases (e.g., are more sensitive to specific SDGs relative to others), and are susceptible to the type and amount of text analyzed. We then show that an ensemble model that pools SDG labeling systems alleviates some of these limitations, exceeding the performance of the individual SDG labeling systems considered. We conclude that researchers and policymakers should care about the choice of the SDG labeling system and that ensemble methods should be favored when drawing conclusions about the absolute and relative prevalence of work on the SDGs based on automated methods.

## Introduction

Humanity is not currently on track to achieve the SDGs by 2030 (Sachs et al. 2022; Usubiaga-Liaño and Ekins 2023; Moyer and Hedden 2020). One factor hampering progress is limited monitoring due to missing data (Dang and Serajuddin 2020; Nilashi et al. 2023). For example, 68% of the environmental SDG indicators lack data (Campbell et al. 2019), and an analysis by the OECD (2020) revealed a similar grim picture for data on gender-related SDG indicators in OECD countries, with 67% of the 104 indicators not having any progress data at all (OECD 2020). As stressed by Nilashi et al. (2023), without such data, “sustainable development is doomed to falter” (p. 2). Given this lack of data, efforts have been initiated to find multiple and novel ways of gathering data on SDG progress that go beyond official statistics, for example through citizen science projects (Venkatesh and Velkennedy 2023; Fraisl et al. 2023). Yet another approach that has increasingly received attention is using textual data as a source for detecting progress on the SDGs, for example, by monitoring scientific publications (e.g., Armitage et al. 2020), patents (e.g., Hajikhani and Suominen 2022), or policy documents (e.g., Tudor et al. 2024).

Scientometric efforts that monitor the SDGs from academic publications have been a main driver of the development of natural language processing methods that allow detection of SDGs in text in the form of specific SDG labeling systems (e.g., Jayabalasingham et al. 2019; Vanderfeesten et al. 2020; Bautista-Puig and Mauleón 2019; Duran-Silva et al. 2019; Sustainable Development Solutions Network 2021; Hajikhani and Suominen 2022). These approaches have led to a number of publications across different disciplines concerned with tracking how SDGs are represented in their respective fields (Mio et al. 2020; Pizzi et al. 2020; Sweileh 2020; Körfgen et al. 2018; Meier 2023; Singh et al. 2023; Purnell 2022). Some efforts have been even more ambitious and have aimed to identify the global academic output with millions of SDG-related publications being categorized and now used by the Times Higher Education Impact Rankings to rank thousands of universities worldwide according to their SDG-related outputs. This illustrates the important role that SDG labeling from text can play in academic research and funding (Smith et al. 2021). Although SDG-related research is not synonymous with progress on the SDGs, an expansion of scientific research is likely needed (Messerli et al. 2019) and conducive to progress in that it fosters evidence-based strategies and innovation (Allen et al. 2021; Schneider et al. 2019; Smith et al. 2018).

The detection of SDGs from text has expanded well beyond the academic literature. Policy documents are a prime example of how textual data can be used to track progress on the SDGs. Indeed, analyzing policy documents with regard to the SDGs may give more specific insight into the extent the SDGs are considered at the implementation level. The promise of the approach is echoed by a number of publications that analyzed policy documents with regard to the SDGs (Forestier and Kim 2020; Meilland and Lecocq 2023; Morita et al. 2020; Xie et al. 2021) and similar efforts using company reporting documents to map SDGs in the business domain (Bose and Khan 2022; Arena et al. 2023).

Although SDG labeling systems that identify SDGs in academic or policy documents hold great promise to help track progress on the SDGs, the increased and widespread use of these systems should be accompanied by efforts to validate the different approaches and ensure that their predictions accurately reflect the SDGs. However, systematic and comprehensive evaluations of the accuracy of these SDG labeling systems are largely lacking. Crucially, the few existing results indicate some striking differences in the predictions of the different SDG labeling systems (e.g., Armitage et al. 2020; Pukelis et al. 2020; Schmidt and Vanderfeesten 2021; Purnell 2022), perhaps due to lack of consensus concerning the relation between science and SDGs (e.g., Rafols et al. 2021). Regardless of the main causes underlying the proliferation of different approaches, an understanding of their differences is key to an informed application of SDG labeling systems. Three key reasons have prevented rigorous comparison of the different SDG labeling systems. First, most SDG labeling systems have been developed for and tested with specific proprietary citation databases, thus limiting their portability to other text sources. Second, there has been a paucity of publicly available data that can be used to validate the predictions of different SDG labeling systems. Third, SDG labeling systems have often been analyzed in isolation without systematic comparisons involving various performance metrics or assessment of biases.

In our work, we aimed to help overcome these limitations by focusing on query-based SDG labeling systems that are widely used and have some desirable properties, such as transparency and interpretability of the queries used to detect SDGs. For this purpose, we relied on our open-source R package, text2sdg (text2sdg.io; Meier et al. 2021), which implements six existing SDG labeling systems, namely, the Aurora (Aurora Universities Network 2020), Elsevier (Jayabalasingham et al. 2019), SIRIS (Duran-Silva et al. 2019), Auckland (Wang et al. 2023), SDGO (Bautista-Puig and Mauleón 2019), and SDSN (Sustainable Development Solutions Network; Sustainable Development Solutions Network 2021) systems and, additionally, OSDG.ai (Pukelis et al. 2022), a publicly available tool. These represent a majority of, albeit not all (cf. Fane et al. 2022), SDG labeling systems currently available for automated detection of SDGs from text. Finally, we also aimed to assess the potential of using an ensemble approach that integrates several of the existing SDG labeling systems. Ensemble models can potentially improve accuracy and generalizability by considering the predictions of multiple models, each of which may have different biases, resulting in a more balanced and representative prediction. Although our work provides a comparison of central text-based approaches already used in practice, we should mention that alternative approaches exist. We discuss some of these and their relative strengths and weaknesses in our discussion.

Our contribution is structured as follows. We first evaluate the categorizations of the SDG labeling systems against those of human experts using labeled data sets covering different text sources, including titles, excerpts of academic publications, and news articles, thus significantly increasing the scope of sources considered in past work (e.g., Armitage et al. 2020; Pukelis et6 al. 2020). Second, we compare the categorizations of the SDG labeling systems to reveal SDG-specific biases, that is, whether different systems tend to make differential predictions for specific SDGs. The potential for bias is particularly important concerning the application of SDG labeling systems to make relative statements about the presence of specific SDGs. For example, the use of biased systems in the detection of some SDGs could lead to an incorrect assessment of investment in some domains (e.g., health) relative to others (e.g., education). Third, we introduce and leverage several novel unlabeled data sets (e.g., Disneyland reviews, cooking recipes, math lectures, random text) to better understand the SDG labeling systems’ susceptibility to the type and amount of text analyzed. Fourth, and finally, we explore whether ensemble-modeling approaches, which aggregate the predictions of several SDG labeling systems to achieve more accurate and robust classification performance, can address some of the potential limitations of individual SDG labeling systems.

**Table 1:** Overview of the SDG labeling systems

Labeling system	SDGs covered	Query operators	Unique keywords per SDG (mean & SD)	text2sdg
Aurora	SDG 1–17	OR, AND, wildcards, proximity search	89.6 (31.6)	$\checkmark$
Elsevier	SDG 1–16	OR, AND, wildcards	74.9 (21.7)	$\checkmark$
SIRIS	SDG 1–16	OR, AND	262 (148)	$\checkmark$
Auckland	SDG 1–16	OR, AND, wildcards	183 (46.5)	$\checkmark$
SDSN	SDG 1–17	OR	62.6 (16.8)	$\checkmark$
SDGO	SDG 1–17	OR	245 (236)	$\checkmark$
OSDG	SDG 1–17	–	–	X

The final column indicates whether the package is included in the text2sdg R package (Meier et al. 2021)

*OR* keywords are combined using logical ORs, implying that only the keywords must be matched to assign an SDG label; *AND* keywords are combined using logical ANDs, implying that multiple keywords must be matched to assign an SDG label; *wildcards* keywords are matched considering different keyword parts; *proximity search* keywords must co-occur within a certain word window to assign an SDG label

## Materials and methods

### SDG labeling systems

Six of the SDG labeling systems analyzed are based on Lucene-style queries (i.e., a query syntax enabling flexible search in text data, from simple keyword search to search involving Boolean operators, wildcards, and other operators), varying considerably in complexity. The SDSN and SDGO systems are least complex because they only make use of OR-operations, implying that they assign an SDG as soon as one of several keywords is matched. The SIRIS and, in particular, the Elsevier and Auckland systems are more complex, as they additionally include AND operations, meaning that multiple keywords must be present to trigger a match. The Aurora system is most complex because it further includes NEAR operations, meaning that keywords must co-occur within a maximum distance to result in a match. These differences in the use of query operators are reflective of the goals with which these queries were designed. For example, the Aurora system draws heavily on the AND operator to minimize false positives (Schmidt and Vanderfeesten 2021). In contrast, the SDGO system leans in the other direction, by only relying on the OR operator and having a large number of keywords per SDG, implying a focus on minimizing false negatives (see Table 1). The OSDG.ai system (Pukelis e4t al. 2022) is based on a machine learning model trained on the OSDG Community Dataset (OSDG 2022). To avoid data leakage and circumvent the OSDG.ai system’s limitation to texts between 50 and 3000 words, we combined the current OSDG.ai system with its less restricted legacy version. For some of these SDG labeling systems (Aurora, Elsevier, and SIRIS), multiple versions exist. We used version 1 from the Elsevier system, version 5.0 from the Aurora system, and version 1.2 from the SIRIS system. We limited ourselves to version 1 from Elsevier because later versions draw on information specific to academic publications and the proprietary Scopus database, thereby limiting its scope. All but the OSDG.ai system are implemented in the text2sdg R package.

#### Labeled data

We used three labeled data sets involving expert assignment of SDG labels to text that were available from different sources (see Table 2).

The *titles* data set (Vanderfeesten et al. 2020) was created to validate the Aurora classification system. The part of the data we used consisted of a survey where respondents had to indicate whether a research paper was relevant for a given SDG. Each of the 244 respondents did this for 100 papers randomly selected from a pool of research papers detected by the Aurora system as SDG relevant. Most papers (95.8%) were rated by a single respondent.

The *excerpts* data set is based on the OSDG Community Dataset (OSDG 2022), which contains tens of thousands of text excerpts from academic articles and policy documents labeled by volunteer respondents. To make this labeling more efficient, the respondents only had to indicate whether or not a suggested label suited the text excerpt. Thus, the respondents simply had to accept or reject a given SDG, but were not asked to select one or more SDGs that might relate to the given text excerpt. Each text was rated by multiple respondents. On average, each text was rated by 6.3 respondents (median = 5). We coded a text as being related to a given SDG if the text received more positive (i.e., related to a given SDG) than negative ratings, which was the case for 82.3% of all texts. The average agreement score as reported by the data set providers is .68 (OSDG 2022), suggesting only moderate agreement between respondents. The survey underlying the OSDG Community Dataset is ongoing, and updated data sets are published regularly. We used the most recent version available at the time of our analysis (Version 2022.10, OSDG 2022).

The *news articles* data set was based on the SDG Knowledge Hub data set (Wulff and Meier 2023), which consists of 9,172 news articles posted on the SDG Knowledge Hub website (sdg.iisd.org). This website was launched in October 2016 and is managed by the International Institute for Sustainable Development (IISD). It hosts news and commentary regarding the implementation of the SDGs. The news articles contain labels that show which SDGs they cover. These labels are assigned by the subject experts who write these news articles and confirmed by SDG Knowledge Hub editors, but there is no quantitative information about the level of agreement between the subject experts and editors.

It is important to note that expert assignment of SDG labels can be subject to random and systematic errors. Random errors can be the product of inattention or selection mistakes and systematic errors of response biases or differences in the interpretation of SDGs. All data sets are likely affected by both errors to some extent. However, random errors are likely lower in the OSDG Community Datasets than in the SDG Knowledge Hub data set and, particularly, the Aurora data set, as the slightly larger number of ratings per text and SDG will have reduced the impact of random errors. Systematic errors, on the other hand, are likely lower in the SDG Knowledge Hub data set in comparison to the other two, as they are strictly provided by domain experts. Notwithstanding these limitations, we consider expert labels the gold standard and treat the expert labels as the criterion to enable a rigorous and standardized evaluation of labeling performance in our analysis.

### Unlabeled data

Three unlabeled data sets were obtained via the kaggle data set search and generated an additional category of unlabeled data of our own (see Table 2). The *Disneyland* data set contains 42,656 reviews of Disneyland locations in Paris, California, or Hong Kong posted by visitors on Trip Advisor (see kaggle.com). The *Cooking recipes* data set contains 82,245 recipes scraped from food-related websites, such as skinnytaste.com (see kaggle.com). The *Math lectures* data set contains 860 lectures posted on YouTube by institutions or creators covering 11 subjects, ranging from algebra to natural language processing, which are related to computer science and mathematics (see kaggle.com). The *Wikipedia* synthetic data sets were generated by concatenating words sampled at random based on the words’ frequencies in the (Wikipedia corpus). The synthetic texts, thereby, reflect the natural word frequency distribution found in natural language. We created synthetic data of different lengths for the analysis of false alarms and to train the ensemble model.

For all four unlabeled data sets, we assumed that SDGs are strictly absent. While this assumption may be incorrect in some cases, it seems likely that there are no meaningful discussions of SDGs in any of the unlabeled data texts.

**Table 2:** Overview of data sets

Data set	Description	# documents	# words/document	Source
Titles	Titles of research papers	10,682	17.8	Vanderfeesten et al. (2020)
Excerpts	Text excerpts from academic articles and policy documents	37,575	94.5	OSDG (2022)
News articles	News articles regarding the implementation of the SDGs obtained from the SDG Knowledge Hub	9172	673.5	Wulff and Meier (2023)
Disneyland reviews	Disneyland reviews from Trip Advisor	42,656	129.7	kaggle.com
Cooking recipes	Recipes from food-related websites	82,245	166.1	kaggle.com
Math lectures	Transcripts of Math lectures posted on YouTube	860	5598.2	kaggle.com
Wikipedia	Synthetic data sets generated by sampling words from the Wikipedia corpus	–	–	Wikipedia corpus

### Metrics

We compared the seven labeling systems quantitatively using a number of common metrics typically used in categorization problems (i.e., sensitivity, specificity, accuracy, F1 score) (see Knox 2018, for an introduction). Sensitivity measures a system’s ability to correctly identify true SDG text sources containing the SDG, whereas specificity measures a system’s ability to identify non-SDG sources not containing the SDG (Hicks et al. 2022). Ideally, SDG labeling systems should be both sensitive and specific, but typically, they trade off against one another. Considering both metrics helps reveal the specific trade-off implied by different systems. We visualize this trade-off by plotting sensitivity against specificity, which is known as the receiver operating characteristic (ROC) (Bradley 1997). The placement of systems along the ROC diagonal indicates the systems’ trade-offs. SDG labeling systems placed closer to the top-right corner, implying a high sensitivity and low specificity, can be considered more liberal, whereas those placed closer to the bottom-left corner, implying a lower sensitivity and higher specificity, can be considered more conservative. Furthermore, the placement of SDG labeling systems along the off-diagonal indicates their overall performance, in terms of an equal weighting of sensitivity and specificity, known as balanced accuracy. In addition, we report two overall measures of performance in accuracy calculated as the number of correct SDG assignments divided by the number of all cases and the F1 score, a popular composite measure combining sensitivity and precision, the latter reflecting the ratio of true positive classifications to all positive predictions (Hicks et al. 2022).

In addition to performance measures, we evaluate the systems’ bias across SDGs. As a technical term, bias refers to a systematic deviation (Dietterich and Kong 1995). We calculate bias for a given SDG as the differences between the number of expert assignments and system assignments normalized by the number of expert assignments. Positive biases, therefore, indicate the tendency of a system to assign a larger number of SDGs than those identified by the experts and vice versa for negative biases.

### Ensemble modeling

To train ensembles of SDG labeling systems, we primarily relied on random forest (R package ranger Wright and Ziegler 2017). Random forest is a common tree-based machine learning algorithm that excels in controlling overfitting and producing accurate out-of-sample predictions, especially in the context of tabular data as present in this analysis (Shwartz-Ziv and Armon 2022). We also evaluated another tree-based algorithm, namely extreme gradient boosting (R package xgboost Chen and Guestrin 2016), but it did not outperform the random forest and, therefore, we do not report the results.

We trained separate models for each SDG predicting the presence or absence of the SDG based on the classifications of the six different SDG labeling systems implemented in text2sdg.io and the number of words in the document texts. The algorithm’s input thus takes the form of a seven-element vector, e.g., [1, 0, 0, 0, 1, 1, 48], containing binary classifications of the SDG labeling systems and an integer for the number of words, while the output is a binary classification of 0 or 1, indicating SDG absent or present, respectively. Including multiple SDG labeling systems as input features allows the algorithm to identify the best way to combine them into an ensemble prediction. Training and evaluation were performed using a repeated k-fold cross-validation procedure. Cross-validation is a technique used to evaluate a model based on its ability to generalize to new data. This is achieved by repeatedly dividing the data into multiple parts (k-folds) and evaluating the models on parts not used to train the model. We trained the models simultaneously on all three expert-labeled data sets (titles, excerpts, news articles) and specifically crafted synthetic data sets (Wikipedia). The synthetic data were generated by concatenating randomly drawn words based on their frequency in Wikipedia. We created the synthetic data to be equivalent in amount and text lengths to the labeled data. To account for differences in the number of texts in the labeled data sets and control the expected model behavior, we used data weights. The data weights control the sampling of cases that are supplied to each of the individual decision trees within the random forest. We weighted the expert-labeled and corresponding synthetic data sets by 1/*N*, with *N* being the number of texts in a data set. This ensures that the three data sources (i.e., titles, excerpts, and news articles) will have equal sway during training. Furthermore, we included an additional factor determining the weight of the synthetic relative to the labeled data. We varied this weight within the range of $w \in [0, 10]$, with a weight of $w = 0$ implying that the synthetic data receive no weight and do not affect training, and a weight of $w = 10$ implying that the synthetic data receive ten times as much weight as the expert-labeled data, impacting training accordingly. Varying the weight of synthetic data in this way creates models that place different emphasis on different types of errors. A model trained with $w = 0$ will focus primarily on avoiding false negatives, because it is effectively only trained on the labeled data that primarily include positive cases, especially the titles and excerpts data. By contrast, a model trained with $w = 10$ will focus primarily on avoiding false positives because the model is trained mostly on the synthetic data, which we assume to be negative cases. Overall, we found the random forest to perform slightly better than the gradient-boosting algorithm; hence, we report the random forest results in the main text.

### text2sdg

To detect SDGs in these different texts, we used the text2sdg R package (text2sdg.io meier2021text2sdg). The text2sdg package provides a common framework for implementing the different systems to detect SDGs in text and makes it easy to quantitatively compare and visualize their results. The text2sdg also makes available the ensemble model presented in this article.

## Results

In the following subsections, we first compare the SDG labeling systems on a variety of metrics on three labeled data sets covering texts from academic publications and news articles. Second, we assess to what extent the different SDG labeling systems show biases concerning different SDGs. Third, we assess the susceptibility of the different SDG labeling systems to produce false positives as a function of the length of the text source in novel unlabeled data sets. Fourth, and finally, we assess the potential of ensemble models that integrate the different SDG labeling systems to address potential limitations of individual SDG labeling systems.

**Fig. 1:**
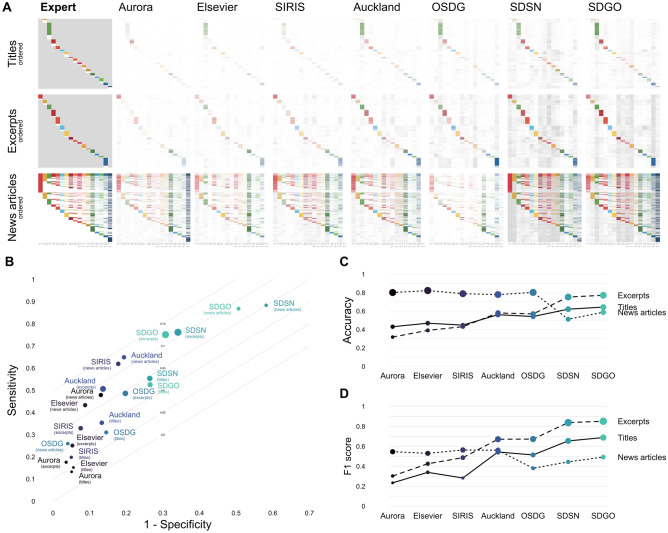
Performance of SDG labeling systems. Panel A shows the SDG labels assigned by experts and seven labeling systems for three data sets consisting of titles of research articles (titles), excerpts of research articles (excerpts), and online news articles (news articles), respectively. The first column shows the labels assigned by experts in the three data sets, with colored lines representing positive ratings, white lines representing negative SDG ratings (experts assigned no SDG), and the gray area representing absent SDG ratings (experts did not rate the SDG; these cases were not considered for the performance evaluation). The remaining columns show the results of the seven labeling systems, ordered by their liberalness. In these columns, the colored and gray lines represent positive SDG predictions, with colored lines representing hits, where the prediction and expert rating agree, and gray lines representing potential false alarms, where the prediction is positive and the expert rating is either missing (titles and excerpts) or negative (news articles). White lines represent negative SDG predictions, irrespective of expert ratings. Panel B shows the performance of the SDG labeling systems across the three data sets by plotting the systems’ sensitivity against 1-specificity, forming an ROC (receiver operating characteristic) plot. This summarizes the performances in two ways. The placement along the off-diagonal reflects balanced accuracy, with the gray dotted lines in the background highlighting several relevant levels. On the other hand, placement along the diagonal reflects liberalness, with those in the top right being more liberal and those in the bottom left being more conservative. Panel C and D show the performance of the SDG labeling systems across the three data sets in terms of accuracy and the F1 score

**Fig. 2:**
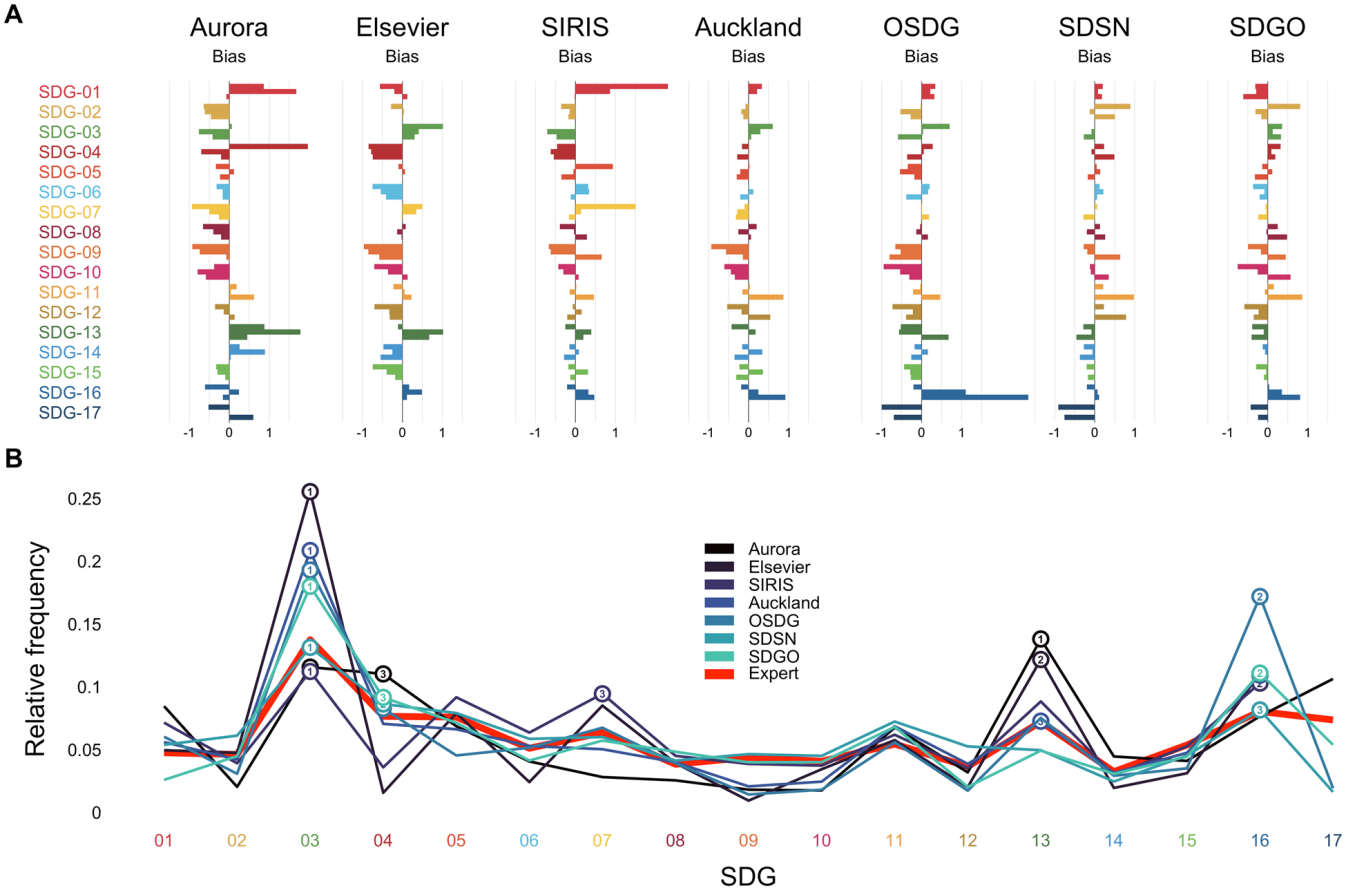
Biases in SDG classifications. Panel A illustrates the biases of SDG labeling systems in classifying each of the 17 SDGs. Biases are calculated as the difference between the observed and predicted SDG proportion for a given data set divided by the observed proportion. A positive bias means that a given SDG was assigned more often than its relative frequency in expert judgments and vice versa for a negative bias. The three vertical stripes for each SDG and system correspond to the titles, excerpts, and news articles data sets. Panel B shows the profile of expert (red) and system labels generated by averaging the relative frequencies of SDGs across data sets. The circles and numbers highlight the three most prevalent SDGs per system

### SDG labeling systems differ in their sensitivity–specificity trade-offs

Our first set of analyses consisted of comparing SDG labeling systems based on predicted labels for documents from three expert-labeled data sets: the Aurora dataset containing ratings of over 10,000 titles of academic publications (*titles*; Vanderfeesten et al. 2020), the OSDG Community Data containing ratings of over 30,000 excerpts of academic publications (*excerpts*; *excerpts* OSDG 2022), and the SDG Knowledge Hub data containing ratings of over 9000 news articles scraped (*news articles*; https://sdg.iisd.org Wulff and Meier 2023). Figure 1 presents the main classification results separately for the three data sets, with panel A showing a breakdown of SDG labels per document. To better assess the differences between each data set, it is helpful to look initially at the first column of each subpanel in Fig. 1A, which contains information on the expert ratings for each data set. For the *titles* data set, texts were only evaluated for a single SDG, and a large majority of documents were judged to contain the SDG (63%). There is no information on whether the texts could reflect additional SDGs; therefore, we ignored these labels in the analysis, indicated by the gray area. Similarly, in the *excerpts* data set, texts were also only evaluated for a single SDG, and a majority of documents were assigned the SDG (82.3%). In contrast, in the *news articles* data set, texts were rated on all SDGs, and all of the documents (100%) were assigned at least one SDG. As a whole, across data sets, only a minority of documents have not been assigned any SDG by the experts, which, as we discuss below, can limit the validation of SDG labeling systems.

The remaining columns in Fig. 1A depict the labels assigned to each document by the SDG labeling systems and, therefore, allow a first comparison between these and the human experts. Visual inspection suggests that all SDG labeling systems showed reasonable accuracy in terms of recovering the “true” SDG labels assigned by experts. This is illustrated by the similar pattern of colored stripes across experts and the SDG labeling systems, which reflect an agreement between experts and SDG labeling systems or, in other words, hits. Nevertheless, the SDG labeling systems showed considerable differences in their ability to detect SDGs, as illustrated by the relative intensity of the colored stripes and gray stripes, reflecting potential false alarms. The results below quantify these differences using common performance metrics.

As can be seen in Fig. 1B, the SDG labeling systems differ substantially in their trade-off between sensitivity and specificity or, in other words, in how conservatively they assign SDGs. This is shown by the fact that their performance—in terms of sensitivity and specificity—varies mostly along the diagonal of the ROC plot. We also observe differences in overall accuracy, but these are not stable across data sets (Fig. 1C), which is due to the different levels of conservatism of the different SDG labeling systems: more conservative ones (e.g., Elsevier) outperform liberal ones for news articles, in which many labels are negative, and vice versa for excerpts and titles, where almost all labels are positive. These patterns hold for the alternative measure of performance, the F1 score (Fig. 1D).

All in all, these results point to SDG labeling systems being differently liberal or conservative; that is, although some SDG labeling systems are very liberal and detect large numbers of SDGs, others are more conservative and assign SDGs to fewer cases. These differences are due to the different queries, but also to the complexity of operators employed (see Table 1). We also find that SDG labeling systems do best in different data sets and it is difficult to identify a single best-performing model. The results point to a limitation of currently available data sets that include only a small proportion of non-SDG-related documents, which introduces difficulties in assessing systems’ susceptibility to producing false positives. We return to this point below when we introduce novel synthetic data sets.

## Biases in SDG labeling systems distort SDG profiles

Biases in SDG labeling systems can lead to inaccurate representation of the prevalence and importance of different SDGs. If an SDG labeling system is more sensitive to certain SDGs than others, it will overestimate the prevalence of those SDGs and underestimate the prevalence of the others. This results in a misleading picture of the work being done to address the SDGs. Biased systems may also create an unfair advantage or disadvantage for certain organizations or groups. For example, if a method is more sensitive to certain SDGs than others, organizations may aim to portray themselves as focusing on those SDGs by relying on such a method. Finally, bias may create confusion or mistrust among stakeholders. If different methods produce significantly different results, it may be difficult for stakeholders to know which results to trust. This can lead to confusion or mistrust in the results, which could ultimately undermine the credibility of the work being done to address the SDGs.

We estimated SDG-specific biases by comparing the relative frequency of SDGs between predicted (i.e., the labels assigned by a given system) and observed labels (i.e., the labels assigned by experts). Specifically, we calculate $bias = \frac{predicted--observed}{observed}$. We then evaluate the profile bias of a given system by correlating the biases across data sets. We also compare the SDG profiles obtained from the SDG labeling systems to the SDG profile of experts. This analysis amounts to assessing the similarity between the profile across SDGs identified by experts and the profile identified by each of the labeling systems.

Figure 2A shows the biases of the different SDG labeling systems. Visual inspection suggests that some biases appear systematically across data sets. For example, considering Aurora, the pattern suggests consistent underestimation of SDGs 2, 6, 7, 8, 9, and 10 across data sets, but overestimation of SDG 13. For Elsevier, one observes consistent underestimation of SDGs 4, 6, 9, 12, 14, and 15, but overestimation of SDGs 3 and 16. We quantified the systems’ average profile biases by correlating the SDG biases between titles and news articles and between excerpts and news articles and computing the average. We did not consider the correlation between titles and excerpts because of a moderate correlation in expert profiles for these two sources. The strongest average profile bias was observed for Elsevier ($\bar{r}=0.72$), followed by OSDG ($\bar{r}=0.39$), Aurora ($\bar{r}=0.38$), SDGO ($\bar{r}=0.31$), SDSN ($\bar{r}=0.2$), Auckland ($\bar{r}=0.08$), and SIRIS ($\bar{r}=-0.05$).

Figure 2B shows that these biases imply substantial differences in SDG profiles derived from the different SDG labeling systems relative to the one derived from experts. This difference is most pronounced for SDG 3 (Good health and well-being). A number of systems overestimate the relative frequency of SDG 3, with Elsevier giving SDG 3 twice as much weight (0.26) as experts (0.13). In turn, SDGs 9 (Industry, Innovation, and Infrastructure) and 10 (Reduced inequalities) tend to receive less weight than that assigned by experts. To further evaluate profile fidelity, we calculated the Spearman’s rank correlation coefficient between expert and system relative frequencies separately for the three data sets. We found Auckland to have the highest average correlation ($\bar{r} =0.76$), followed by SDGO ($\bar{r}=0.72$), OSDG ($\bar{r}=0.71$), Elsevier ($\bar{r}=0.70$), SIRIS ($\bar{r}=0.67$), SDSN ($\bar{r}=0.63$), and, finally, Aurora ($\bar{r}=0.57$).

To summarize, we find that SDG labeling systems appear to have different biases, with a number of these underestimating or overestimating the presence of specific SDGs relative to experts. These biases can be important for the relative assessment of work on the SDGs and emphasize that the SDG labeling systems should not be used interchangeably.

**Fig. 3:**
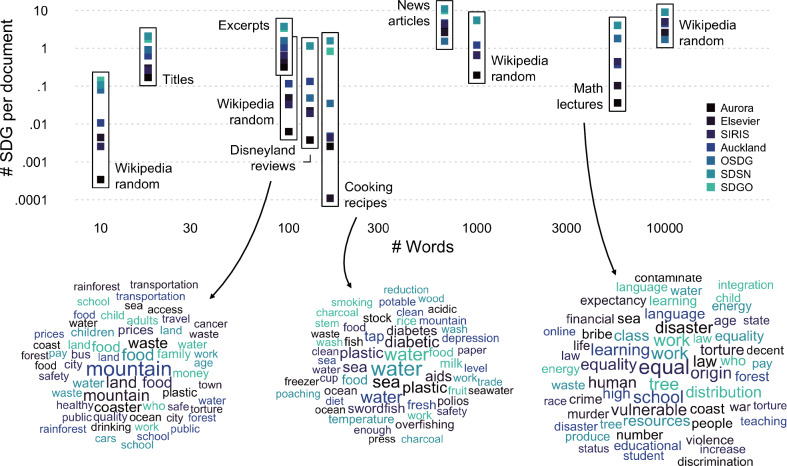
False-positive predictions of SDG labeling systems. The figure shows the number of SDGs assigned by the seven SDG labeling systems to three natural language data sets unrelated to SDGs (Disneyland reviews, cooking recipes, and math lectures), four synthetic data sets of different lengths created from Wikipedia word frequencies (Wikipedia random), and three expert-labeled data sets (titles, excerpts, news articles), as a function of the number of words per document. The word clouds at the bottom consist of the keywords that triggered the assignment of SDGs for the three natural language data sets, with size coding the frequency of keyword hits and the color coding the SDG labeling system

**Fig. 4:**
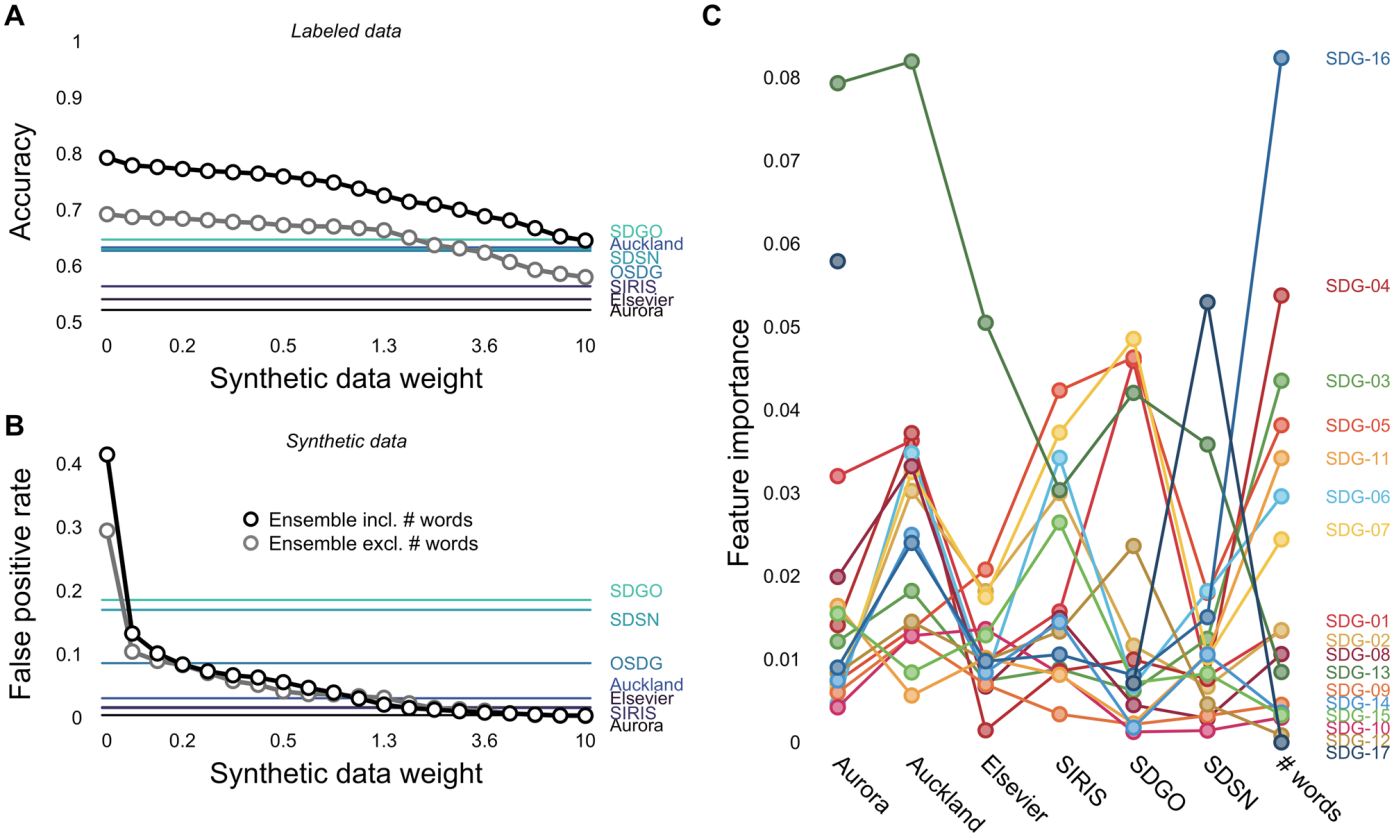
Ensemble model performance. Panel A and B illustrate the performance of ensemble models drawing on the classifications of the six SDG labeling systems relative to the SDG labeling systems on their own. Panel A shows the accuracy for the three expert-labeled data sets as a function of the amount of training weight given to synthetic non-SDG documents generated from Wikipedia word frequencies. Panel B shows the false-positive rate as a function of the training weight. In both panels, the black line shows the performance of an ensemble model that includes document length as a feature, whereas the gray line corresponds to a model that does not include document length. Panel C shows the SDG-specific feature importance for the ensemble model that includes document length and has been trained with a non-SDG data weight of 1

### SDG labeling systems can produce many false positives when applied to large text sources

Existing validation data sets—such as those used above to test the accuracy of SDG labeling systems—may not accurately reflect the performance of SDG labeling systems in many real-world applications that involve larger samples of text. As mentioned above, existing data sets only include a small proportion of documents unrelated to the SDGs, which can lead to an overestimation of the accuracy of the SDG labeling systems relative to applications in which they are tested on a diverse range of documents that can potentially contain many false positives. To investigate this, we conducted evaluations of the SDG labeling systems using data sets that are ostensibly unrelated to SDGs. Although these data sets may include some references to SDG-related topics, the comparison of domains and lengths of text can be helpful in providing an understanding of the systems’ relative tendency to produce false positives across contexts.

In this analysis, we used existing and synthetic data sets; specifically, three natural language data sets consisting of Disneyland reviews ($N = 42,656$), cooking recipes ($N = 82,245$), and math lectures ($N = 860$). In addition, we generated synthetic texts by sampling from a word frequency list derived from Wikipedia and generating documents containing 10, 100, 1000, and 10,000 randomly sampled words.

Figure 3 presents the results of the SDG labeling systems for the various data sets. The plot shows the number of SDGs identified in each document by each SDG labeling system, plotted against the average length of the documents in the respective data set. The plot includes data from the novel data sets (those likely not related to the SDGs), as well as the three expert-labeled data sets. We use the number of SDGs per document as a proxy for the false-positive rate. Consequently, by comparing the number of SDGs identified by the SDG labeling systems across different data sets and document lengths, we hope to understand the susceptibility to producing false positives and identify any patterns or trends in labeling accuracy as a function of document length.

Several noteworthy results emerge. First, all SDG labeling systems produce false positives for all types of data sets. Second, the tendency of SDG labeling systems to produce false positives is in line with their level of conservatism, which we discussed above. For example, Aurora and Elsevier appear to be very conservative in identifying SDGs, leading to an overall low false-positive rate. Third, across all SDG labeling systems the tendency to detect SDGs increases considerably when the length of the texts increases. Specifically, whereas some SDG labeling systems produced between 0.0003 (Aurora) and 0.144 (SDGO) for synthetic texts of length 10, systems produced between 1.71 (OSDG) and 9.03 (SDSN) SDGs per document for synthetic texts of length 10,000.

The word clouds illustrate the keywords that triggered the assignment of SDGs for the three natural language data sets (Disneyland reviews, cooking recipes, math lectures). As can be seen, the keywords relate to many frequent topics that fit the respective SDGs but also the mundane contexts of the three data sets. These word clouds can be seen to highlight the limitations of query-based approaches that do not control for the frequency of keywords in natural language nor the context in which they occur.

### Trained ensemble models alleviate the shortcomings of existing labeling systems

Previous sections illustrated serious shortcomings of existing SDG labeling systems. SDG labeling systems differ more in conservatism than in accuracy; they have considerable SDG-specific biases, and they commit many false positives when the length of documents increases. However, these shortcomings did not affect systems uniformly—some were more prone to bias, whereas others were more prone to false alarms. We leverage this fact to train ensemble models of SDG labeling systems.

To train ensemble models, we rely on standard tree-based machine learning algorithms, which are known to perform well for tabular data (Shwartz-Ziv and Armon 2022). We report the result of random forest models that integrate the predictions of the six publicly available SDG labeling systems implemented in text2sdg and document length as input features. We train these models based on the expert-labeled data and matching synthetic data, which helps to control for excessive positive rates in the labeled data and produce variants with trade-offs between false negatives and false positives. We report the systems’ accuracy, F1 score, and false-positive rate in out-of-sample prediction based on repeated cross-validation.

Figure 4 shows the performance of two groups of ensemble models that either included (black) or excluded (gray) document length as a predictor in comparison to the six individual SDG labeling systems integrated within the ensemble model and the OSDG system. We find that for a wide band of moderate synthetic data weights, the ensemble model achieves a higher average out-of-sample accuracy than all individual SDG labeling systems, while committing only as many false alarms as the most conservative ones. Furthermore, we find that including document length as a predictor substantially improves the performance of the ensemble model.

We analyzed whether the ensemble model suffers from the same sensitivity–specificity trade-off and found that this was not the case. Specifically, for a synthetic data weight of 1, we find that the ensemble model is on a par with the best-performing conservative model for the news article data (Ensemble: $Accuracy =0.83$, $F1 =0.54$; Elsevier: $Accuracy\,0.82$, $F1 =0.46$) and also with the best-performing liberal system for the titles and excerpts data (Ensemble: $Accuracy =0.69$, $F1 =0.71$; SDSN: $Accuracy =0.69$, $F1 =0.73$), implying an even performance across data sets. We also evaluated the profile bias and fidelity of the ensemble model. We observed a profile bias at the lower end of the individual systems ($\bar{r} =0.14$) and a expert profile fidelity far outperforming the individual systems ($\bar{r} =0.92$).

The ensemble model can further be used to understand the usefulness of the individual SDG labeling systems through an analysis of feature importance. Feature importances were determined separately for each SDG by permuting individual predictors and recording the drop in performance. Higher drops imply larger feature importance. Figure 4C shows the permutation-based feature importance separately for the different SDGs. It can be seen that importance varies considerably across SDGs, which highlights not only differences in the quality of SDG labeling systems across the SDGs but also the lack of comparable data on the different SDGs. Despite the considerable variance across SDGs, the ensemble model preferred to rely on some systems more than others. Specifically, Auckland, SDGO, and SDSN received higher feature importance relative to the Aurora, Elsevier, and SIRIS systems.

## Discussion

A number of automated SDG labeling systems have been proposed to identify work on the Sustainable Development Goals (SDGs) from text, and the recent uptick in publications that make use of these systems highlights the promise of this approach. In this work, we aimed to systematically validate and compare SDG labeling systems. Systematic comparison of SDG labeling systems, including their relative performance and potential biases, is a crucial step toward identifying the most reliable and accurate tools and, furthermore, building confidence in the use of automated methods for monitoring work on the SDGs.

### Main findings and implications

We compared seven SDG labeling systems using a variety of text sources—including research papers, news articles, and non-SDG-related texts—and a variety of metrics. Our results suggest that the existing SDG labeling systems differ in accuracy and that their performance varies considerably across text sources. These performance differences are due to differences in specificity (true-positive rate) and sensitivity (true-negative rate). Additionally, the SDG labeling systems have different biases that can have an impact on the overall profile of the SDGs identified, with some emphasizing specific SDGs (e.g., health) relative to experts. Our finding of biases is important because it reveals a potential for misleading representation of work on the SDGs that could create confusion about the relative investment in different SDGs and even undermine trust in the use of automated methods. Some researchers have pointed out how institutional rankings using several, often non-transparent criteria can lead to a lack of convergent validity between rankings and associated confusion (Berg et al. 2022). The increasing reliance on automated systems for ranking institutions’ contributions to the SDGs requires reliable systems that aim to reduce bias. Finally, and more broadly, these results suggest that SDG labeling systems should not be used interchangeably.

One alternative to the use of single SDG labeling systems is the use of ensemble models that pool multiple labeling systems. Our results suggest that ensemble models can overcome some of the limitations of individual labeling systems. In particular, our results suggest that an ensemble approach is able to achieve higher performance compared to existing systems, and is less susceptible to biases and variations in the type and amount of text analyzed. All in all, these results suggest that ensemble models may be a good alternative to existing systems to detect SDGs in an automated fashion. Our ensemble model is freely available in our text2sdg R package.

It is important to note that the appropriateness of a given system will depend on the specific use case. For example, if the goal is to maximize the detection of SDG-related texts (i.e., high recall/sensitivity), a more liberal approach might be appropriate. This could be sensible in a scenario where the a priori likelihood of a text being related to the SDGs is high (e.g., mission statements of nonprofit organizations, Tudor et al. 2024). In turn, if one wants to ensure that no texts are falsely identified as being related to an SDG (i.e., false positives), a conservative approach might be the better choice. However, our results suggest that the ensemble model successfully combines the best of both worlds, making it objectively the best choice across the use cases considered. The performance of the ensemble model is on par with the best-performing conservative model for the news article data and with the best-performing liberal systems for the titles and excerpts data. The ensemble model thus achieves an even performance across data sets (i.e., use cases) while also demonstrating a low profile bias and an unmatched expert profile fidelity. Furthermore, the ensemble model could still be tailored to a given use case by adjusting the conservativeness of the model by adjusting the weight of the synthetic data that the model was trained on. Future work will be needed to assess whether these advantages generalize broadly to other use cases.

### Limitations and future directions

We would like to point out several limitations of our work as well as some opportunities for future research in the field of automated SDG labeling. First, concerning data limitations, a general limitation of current labeled data sets is that labels were produced by single judges (Aurora), have unknown level of agreement between multiple judges (SDG Knowledge Hub), or show less than perfect agreement between judges (OSDG Community Dataset). Limits to label validity is a problem for any labeling system that relies on these data for training or validation and therefore a main issue to address in future efforts. Furthermore, none of the expert-labeled data sets used in our work allow for a good estimation of false positives, as these documents were not randomly or representatively sampled from the respective class of documents. We alleviated this problem by relying on synthetic data sets that were unrelated to SDGs but this approach does not account for the word co-occurrence patterns of actual negative SDG texts. Future work should focus on producing better and larger validation data sets, comprising ratings from multiple judges, that can be used to further train and validate SDG labeling systems, ideally also from domains beyond academic papers or news articles, if these methods are to be validated beyond these domains, for example, concerning policy documents.

Second, although we compared seven SDG labeling systems that represent the majority of the existing systems for automated detection of SDGs from text, there are additional systems that could be considered. However, some of these offer queries for only a subset of SDGs (e.g., Armitage et al. 2023), involve proprietary systems (Dimension.ai; Fane et al. 2022), or, as in the case of novel versions from Elsevier, were developed and optimized for use in specific databases. The proprietary nature and optimization for specific databases render them difficult to implement and make them available to a wider audience through systems such as text2sdg. Overall, we believe that making such automated tools publicly and easily available would be an important step toward assessing and improving automated labeling of SDGs and, ultimately, making a contribution to sustainable development.

Third, although the ensemble approach used in this study was effective in addressing the shortcomings of existing SDG labeling systems, it is still limited by the keywords used with the included systems. These keywords were selected using procedures specific to certain data sets, and it is likely that the current keyword set is incomplete for some or all of the SDGs. Once larger, more diverse, and representative expert-labeled data sets become available, it would be beneficial to learn relevant keywords from these expert labels that could further help improve an ensemble approach.

Fourth, our work compared mostly query-based systems because they are widely used, interpretable (i.e., queries can be shared transparently and discussed), computationally cheap to implement, and, at least for those used in our work, publicly available for open use. Recently, other promising approaches have emerged that use transformer-based models to detect SDGs in text that may also offer some advantages (e.g., Guisiano et al. 2022; Mandilara et al. 2023; Morales-Hernández et al. 2022). For example, Mandilara et al. (2023) trained several different transformer models on the OSDG data and achieved very high classification performance, albeit there is reason to believe that this high performance is partly due to a priori selection of texts and other analytic choices that do not allow a direct comparison to our results. One possible advantage of transformer-based models is the ease of use across several languages and such methods have already been adopted to detect SDGs in non-English texts (e.g., Japanese; Matsui et al. 2022). Furthermore, these models can be fine-tuned in an iterative fashion to specific domains or languages, which can help to improve their performance and accuracy in specific contexts (Hajikhani and Cole 2023; Hussain et al. 2023). Some efforts in this direction have also been based on queries rather than expert-labeled data (Vanderfeesten 2022); thus, developing queries and data may remain a priority even when relying on transformer architectures. All in all, as natural language methods evolve, so will approaches to detecting SDGs from text. However, we suspect that the availability of high-quality labeled data free of the issues of current data, privacy issues associated with using services from commercial providers (e.g., OpenAI), and inherent limitations in interpretability associated with large language models may still favor the use of query-based systems that can be more easily deployed locally and are more transparent. Future work will need to assess whether such advantages come at a serious cost to accuracy.

Fifth, and finally, we should make clear that our approach and any text-based approach should be careful not to confound the detection of SDG-related text from actual progress on the SDGs. Of course, as many text sources are collated and compared, there is hope for identifying true signals of progress from textual data. Nonetheless, and although we see promise in adopting text-based approaches for the monitoring of sustainability efforts, we also see much-needed work in establishing a solid link between the linguistic expression of SDG efforts and their real-world implementation.

## Conclusions

In conclusion, we presented a comparison of several SDG labeling systems to identify work on the Sustainable Development Goals (SDGs) in text sources. Our approach was based on the use of novel data sets and several performance metrics. We found that current SDG labeling systems suffer from several shortcomings, but that ensemble modeling techniques allow us to overcome some of the limitations of existing approaches. We demonstrate that an ensemble approach is able to achieve higher specificity and sensitivity compared to existing SDG labeling systems, and is less susceptible to biases and variations in the type and amount of text analyzed. Our findings have important implications for researchers and policymakers seeking to accurately monitor progress on the SDGs, and we recommend the use of ensemble approaches as best practice when drawing conclusions about the absolute and relative prevalence of work on the SDGs based on automated methods.

## Data Availability

The text2sdg R package is available on the Comprehensive R Network (https://CRAN.R-project.org/package=text2sdg). All data sets used in this analysis are publicly available. Links are included in the main text.
